# Estrogens, Estrogen Receptors and Tumor Microenvironment in Ovarian Cancer

**DOI:** 10.3390/ijms241914673

**Published:** 2023-09-28

**Authors:** Marta Justyna Kozieł, Agnieszka Wanda Piastowska-Ciesielska

**Affiliations:** 1 Department of Cell Cultures and Genomic Analysis, Medical University of Lodz, 90-752 Lodz, Poland; agnieszka.piastowska@umed.lodz.pl; 2 BRaIn Laboratories, Medical University of Lodz, 92-216 Lodz, Poland

**Keywords:** estrogens, estrogen receptors, ovarian cancer, proliferation, tumor microenvironment

## Abstract

Ovarian cancer is one of the most common cancers in women and the most concerning issues in gynecological oncology in recent years. It is postulated that many factors may contribute to the development of ovarian cancer, including hormonal imbalance. Estrogens are a group of hormones that have an important role both in physiological and pathological processes. In ovarian cancer, they may regulate proliferation, invasiveness and epithelial to mesenchymal transition. Estrogen signaling also takes part in the regulation of the biology of the tumor microenvironment. This review summarizes the information connected with estrogen receptors, estrogens and their association with a tumor microenvironment. Moreover, this review also includes information about the changes in estrogen receptor expression upon exposition to various environmental chemicals.

## 1. Introduction

Ovarian cancer (OC) is one of the most common cancers in women in developing countries. In recent years, it has been postulated that OC is one of the most concerning issues in gynecological oncology, causing more than 200,000 deaths worldwide in 2020 [1]. Due to a late diagnosis, treatment is challenging and the 5-year survival rate is still under 50%. As in every cancer, when OC is diagnosed early, most women may be treated effectively using standard therapeutic approaches. Unfortunately, once OC spreads to the pelvic and abdominal organs or/and beyond the peritoneal cavity, treatment becomes much more difficult. OCs are a molecularly truly diverse group of cancers. They may be classified based on their origin into epithelial, stromal and germ cells. Most ovarian neoplasms are of an epithelial origin. Another classification, based on histology and carcinogenesis, includes high-grade and low-grade ovarian neoplasms. The stage of ovarian cancer can also be presented on the FIGO scale, where stage I is the least advanced disease and stage IV is the most advanced disease [2]. Treatment of ovarian cancer mostly depends on surgery and chemotherapy treatment. Recently, it has been shown that usage of both 4-Hydroxy-Tamoxifen (4-OHT) and Gatipotuzumab has better outcomes, and may increase the efficacy of treatment [3]. This discovery seems to be important since the authors noticed that there is an association between TA-MUC1 and estrogen receptors (ERs) [3]. Although many research studies have been carried out on ovarian cancer, a detailed molecular mechanism has not been revealed yet. As far as we know, the expression of GPER1 in ovarian cancer tissues is not clear. To achieve a better understanding, and then better treatment, it is necessary to have a better knowledge about OC, especially in the case of both classical and non-classical ERs and tumor microenvironment (TME). Therefore, the purpose of this review is to summarize recent literature concerning ERs, the involvement of TME regarding them and the progression of OC.

## 2. Estrogens, ER Expression and Signaling Pathways

**Figure 1:**
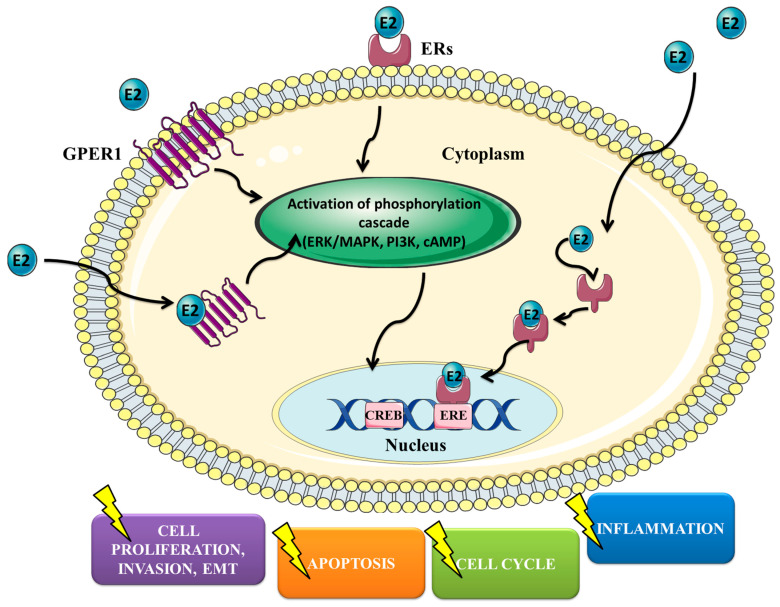
Main mechanisms of estrogen-induced change in the biology of ovarian cancer cells. E2—estradiol, ERs—estrogen receptors, EMT—epithelial to mesenchymal transition, GPER1—G-protein-coupled estrogen receptor 1, ERE—estrogen response element, CREB—cAMP-response-element-binding protein.

It is known that estrogens modulate both physiological and pathophysiological processes [4]. Nevertheless, they also have a vital role in the functioning of the cardiovascular and immune systems [5,6]. The ovaries are the central reproductive organs of women that produce hormones such as testosterone, progesterone and estrogen [7]. Hormones produced by the ovaries play a vital role not only in the ovaries but also in the endometrium, where they stimulate changes in the endometrium during periodic changes [8]. Therefore, proper functioning of the ovaries is necessary for, among others, maintaining pregnancy. Among estrogens, we can distinguish estrone (E1), 17β-estradiol (E2) and estriol (E3), of which E2 is the most dominant estrogen [4]. It was observed that estrogens may stimulate the growth of ovarian cancer cells in vitro [9]. For example, Matsumura et al. showed that E2 stimulates the proliferation of Caov-3 and OVCAR3 cells via Akt/ERK cascades [10]. Interestingly, it was noticed that hormone replacement therapy (HRT) in postmenopausal women is associated with increased incidence and mortality of OC [11]. Therefore, the question was whether physicians should treat women after resection of ovaries with HRT. Fortunately, Eeles et al. stated that women after surgical treatment of OC can safely take HRT, to help them overcome inconvenience associated with the resection of the ovaries and lack of production of hormones [12]. It was also presented that HRT administration is related to the reduced risk of colorectal cancer (CRC) incidence [13]. In turn, it was observed that HRT increases the recurrence of breast cancer (BC) with positive hormone receptors [14]. Side effects of HRT were also linked with cardiovascular diseases [15]. Nevertheless, the statement regarding HRT and coronary heart disease has changed in recent years [16]. Although the role of HRT and its negative impact on women’s health have been discussed for several decades, no clear conclusions have yet been drawn. Recent results are hopeful, suggesting that HRT does not have as many negatives as initially thought, and significantly increases the quality of life. Cellular signaling of estrogens is mediated via ERs. ERs are a family of transcription factors that control the biological function of estrogens via regulating gene transcription through estrogen response elements (EREs). Estrogens may modulate the biology of cancer cells by affecting processes like cell proliferation, invasion, apoptosis, cell cycle and inflammation (Figure 1).

**Figure 2:**
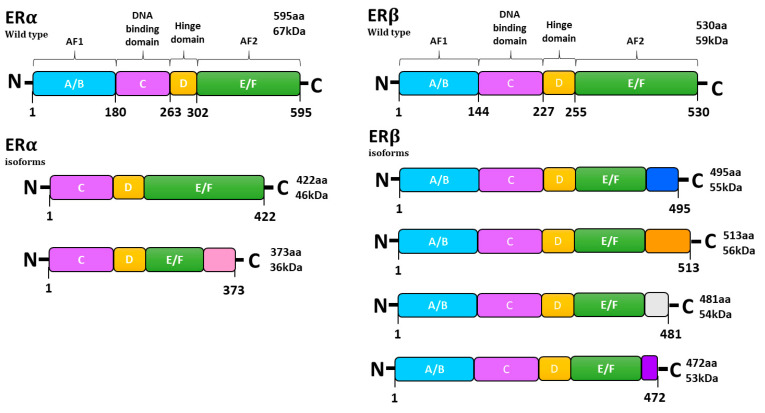
Structure of estrogen receptor α and estrogen receptor β and their isoforms. ERα—estrogen receptor α, ERβ—estrogen receptor β, AF1—activation function 1, AF2—activation function 1.

Among ERs, we can distinguish both classical (ERα and ERβ) and non-classical (G-protein-coupled estrogen receptor 1 (GPER1)) receptors. Research on the expression of classical ERs seems consistent and we can come to conclusions. The ratio between estrogen receptors (ERα and ERβ) has a significant role in ovarian cancer development [17]. Together with the progression of ovarian cancer, the expression of ERα increases, while the expression of ERβ decreases. Nevertheless, in the case of GPER1, it is difficult to draw clear conclusions. On the one hand, some studies reported that GPER1 has a potentially tumor-promoting role in OC and may predict lower patient survival [18,19]. Its expression was also observed to be correlated with the histological grade of the OC. On the other hand, others have not observed any correlation between GPER1 expression and clinical stage and/or patient survival or even GPER1 expression being correlated with higher survival [20,21,22]. Because studies are not consistent, it seems that more studies should be performed to obtain confirmation. ERα and ERβ are encoded by *ESR1* and *ESR2* genes, respectively. *ESR1* is located on chromosome 6 (6q25.1), while *ESR2* is on chromosome 14 (14q23.2) [23]. Both estrogen receptors were found to be located in the cell membrane, but they were also found in cytoplasmic organelles, including mitochondria and the endoplasmic reticulum [24]. ERs consist of six domains: A/B, C, D and E/F starting from amino and ending at carboxyl terminals (Figure 2). The wild type of ERα and ERβ protein has a mass of 66 and 59 kDA, respectively. The A/B domain is responsible for the specificity of the receptor, the C domain is the DNA-binding site and the D domain connects the C domain with the E domain and stabilizes the binding to the DNA in the C domain. The E/F domains are responsible for binding to the ligand. AF1 and AF2 are mandatory for the activation of both ERα and ERβ. GPER1, also known as GPR30, is a non-classical estrogen receptor encoded by the *GPER* gene that is located on chromosome 7 (7p22.3). Normally, GPER1 is present in the endoplasmic reticulum; however, its presence was also observed in the plasma membrane [25]. It was proved that GPER1 binds with high affinity with estradiol. After binding, GPER1 is responsible for the rapid activation of numerous signaling pathways. It promotes the production of cAMP and activation of the epidermal growth factor receptor (EGFR) and it affects signaling pathways like PI3K/Akt and ERK/MAPK [25,26].

## 3. Ovarian Cancer Proliferation, EMT and Cell Invasiveness

ERα was reported to modulate the expression of genes associated with cell proliferation and tumor growth in epithelial ovarian cancer [27]. In turn, Bossard et al. showed that mice with increased expression of ERβ have reduced tumor growth [28]. Moreover, increased expression of ERβ in BG-1 cells significantly decreased cell proliferation stimulated with estradiol [28]. Similar results were obtained in other cell lines, like breast and prostate cancer, where transfection of ERβ decreased motility and invasion of cells [27]. Therefore, it is believed that ERα has pro-cancerous abilities, while ERβ is anti-cancerous, which underlines that this disproportion may have a crucial role in ovarian carcinogenesis [28,29,30]. It is well known that estrogens stimulate proliferation, while anti-estrogen drugs abolish the proliferation of ovarian cancer both in vitro and in vivo [27]. Their ability to enhance the proliferation of neoplastic cells along molecular pathways may occur in a receptor-dependent and receptor-independent manner [31]. The first way is strongly associated with the ERα receptor. After binding estrogen with ERα, a signal cascade is triggered, which causes increased transcription of genes associated with cancer progression such as c-fos, c-myc, growth factors and cyclins that regulate cell cycle progression [31]. Migration of ovarian cancer cells and epithelial to mesenchymal transition (EMT) may be also stimulated with estrogens acting via ERα by decreasing the expression of E-cadherin and increasing EMT-related transcription factors: Snail and Slug [32]. It was also postulated that estrogens may affect adhesion to extracellular matrix proteins via ERα [30]. Increased expression of ERβ resulted in a decreased number of cells in the S phase and an increase in G2/M in a BG-1 cell line. Regulation of cell cycle progression was also seen through cyclin D1 and A2 modulation. Moreover, ERβ was found to modulate total retinoblastoma (Rb), its phosphorylated form, pAkt, cyclin D1 and A2 [28]. Also, ERβ was proposed to modulate the expression and activity of ERα; therefore, its clinical utilization may be worth it [28]. In another study, in a different cell line, the antitumoral effect of ERβ, however, was independent of ERα and estradiol [28]. The discrepancies between the cell lines may be due, among others, to the different mutations that occur in them. In OVCAR3 and OAW-42 cells, the usage of four different ERβ agonists resulted in decreased proliferation [33]. According to the previous results, the knockdown of ERβ stimulated the growth of OAW-42 cells [33]. Schüler-Toprak et al. also showed that the acting of the ERβ agonists is related to β-catenin (CTNNB1) and amyloid β precursor protein (APP) in OAW-42 cells. In SKOV3 cells, increased expression of ERβ resulted in decreased growth and migration [34]. The authors observed that these effects were associated with the modulation of cyclin-dependent kinase inhibitor p21 (WAF1), cyclin A2 transcripts and fibulin 1c [34]. Banerjee et al. showed that activation of ERβ with a newly developed agonist (OSU-ERb-12) abolishes the ability to grow and migrate and invasiveness of ovarian cancer cells [35]. Because EMT is strongly associated with the migration and invasion of cancer cells, an observation considering the role of ERα and ERβ has also been made. It was shown that increased expression of ERβ results in increased E-cadherin (E-cad) and decreased Snail expression [35]. At the same time, it was proved that ERβ agonists may downregulate stemness markers like SOX2, Oct4 and Nanog. Non-genomic effects that may stimulate cell proliferation rely on binding to GPER and thus induce extracellular-signal-regulated kinase (ERK), phosphoinositide 3-kinase (PI3K) and epidermal growth factor (EGFR) signaling [31]. Modulation of GPER1 has been shown to affect ovarian cancer cell growth. Yan et al. showed that the selective GPER-1 agonist (G-1) in a dose of 10 nM increases migration and invasiveness of an ERα-negative cell line via promotive production and activation of MMP-9 [36]. Knockdown of GPER-1 agreed with previous results—it resulted in a reduction in migration and invasion [36]. Changes in invasion, proliferation and migration also affected another cell line, SKOV3, where GPER1 knockdown resulted in their decrease and was also associated with changes in the expression and activity of MMP-2 and MMP-9 [37]. Additionally, Yan et al. presented that GPER1 may modulate the expression of ERα and ERβ [37]. In turn, it was also shown that usage of G-1 may decrease proliferation and induce G2/M cell cycle arrest [22]. It was presented that G-1 promotes the activation of mitotic-promoting factor (MPF) and phosphorylation of nuclear mitotic apparatus protein 1 (NuMA) [38]. Treatment with G-1 also causes a decrease in the number of cells in the G1 phase, an increase in prophase and a decline in metaphase, anaphase, telophase and cytokinesis. Wang et al. showed that G1 inhibits the proliferation of SKOV3 and IGROV-1 cell lines in a dose-dependent manner and that it disrupts the morphology of these cells [38]. The differences between these works may be associated with different doses used for experiments.

## 4. Interaction of Environmental Chemicals, Estrogen Receptors and Ovarian Cancer Proliferation

Some substances present in the environment as pollution, food/cosmetic additives and many others may have an impact on the hormone-dependent tissues, due to the structural and functional similarity to naturally occurring estrogens. Substances like these are called xenoestrogens and may directly and/or indirectly bind with ERs and thus change the biology of cells. Some of these compounds may be of a natural origin, others synthetic. Genistein belongs to a subgroup of isoflavones (the flavonoid family), at physiological concentrations, activates the nuclear estrogen receptors ERα and ERβ and affects TGFβ signaling pathways [39]. Genistein is also the most common natural substance with estrogen activity. Chan et al. showed that genistein and daidzein (another member of the isoflavone family) suppress the proliferation, motility and invasiveness of ovarian cancer cells via modulation of the expression of ERβ [40]. Moreover, they also observed that changes in the behavior of cells were associated with increased expression of p21 and E-cad, and with decreased expression of vimentin (VIM). The modulation of PI3K/AKT signaling was also described [40]. A similar observation has been made for resveratrol, a naturally occurring phytoestrogen, with downregulation expression of ERα IGF-1R, p-IRS-1, p-Akt1/2/3 and cyclin D1 [41]. Sang et al. showed that bisphenol A (BPA) induces the proliferation of ovarian cancer cells via the regulation of matrix metalloproteinase-2 (MMP-2), matrix metalloproteinase-9 (MMP-9) and intercellular cell adhesion molecule-1 (IMAC-1), but the addition of an ERα inhibitor abolished this effect, suggesting that BPA promotes ovarian cancer cells via the ERα signaling pathway [42]. This statement seems to be confirmed by Sang et al. The authors showed that proliferation, migration, invasion and adhesion stimulated with BPA in OVCAR3 cells depend on the activity of ERα [42]. Hwang et al. showed that genistein can reduce BPA-stimulated proliferation in BG-1 cells [43]. Moreover, Hwang et al. also presented that the mechanism is associated with the regulation of cell cycle progression [41]. Liu et al. observed that histamine in a dose of 50 ng/mL induces the proliferation of OVCAR3 cells after 48 h by regulating the expression of both ERα and ERβ [44]. The same team also observed that apigenin, a natural flavonoid found in many plant species, inhibits histamine-induced proliferation via the PI3K/AKT/mTOR pathway [44]. Similar results were obtained in other cell lines like cervical cancer or breast cancer [44,45]. The PI3K/AKT/mTOR pathway was also involved in the regulation of apoptosis and autophagy with Tanshinone I (Tan-I), an extract from the Chinese medicinal herb Salvia miltiorrhiza Bunge [46]. In our study, we observed that estrogenic mycotoxin (Alternariol; AOH) stimulates apoptosis in ovarian cancer cells via ERα and also modulates migration, proliferation and invasion [47]. Nevertheless, the effect on the expression of ERs in both studies has not been shown. Aconitine, a substance produced by plants, has also been described in the context of ovarian cancer. Wang et al. presented that aconitine decreased cell viability, colony formation and migration of A2780 cells. The same team also showed that treatment with aconite induces DNA damage and apoptosis in cells via regulation of the expression of ERβ and also other factors connected with estrogen signaling like vascular endothelial growth factor (VEGF) and hypoxia-inducible factor 1α (HIF1α) [48]. Ataei et al. showed that cadmium chloride induces the proliferation of ovarian cancer cells by affecting the expression of ERs and then activation of the ERK1/2/MAPK pathway and c-jun, c-fos and foxo3a transcription factors. It was also shown that the cadmium acting via ERs may affect the expression of progesterone receptors [49]. Some compounds were also investigated taking into consideration non-classical estrogenic pathways. Hoffmann et al. showed that tetrabromobisphenol A (TBBPA) stimulates the proliferation of OVCAR3 and KGN cells via the GPR30 pathway [50]. The addition of a GPER1 antagonist reversed this effect [50]. Summary information regarding the regulation of ERs expression is presented in Table 1.

## 5. Tumor Microenvironment (TME) in the Progression of Ovarian Cancer 

In recent years, non-cancerous cells constituting TME are believed to be critical mediators of tumor progression. Moreover, the importance of the interplay between tumor cells, stromal cells, immune cells and extracellular molecules in TME is emphasized as it has a profound effect on antitumor immunity and immunotherapeutic response [52]. The importance of TME is emphasized with the fact that it is chosen as a therapeutic target in cancer treatment and enjoys great interest in both research and clinical trials [53]. Nevertheless, one of the main difficulties in targeting the TME in cancer therapy is that host cells or non-cellular components of the TME may have different associations with tumor cells [53]. Therefore, further research on the TME in cancers is necessary because there is a lack of in-depth knowledge about these cells and their interactions that results in failed therapy. As it was proved, each cancer is different in terms of cells that belong to TME; however, we can distinguish cell types that are always included in TME: cancer-associated fibroblasts (CAFs), tumor-associated macrophages (TAMs), myeloid-derived suppressor cells (MDSCs), lymphocytes T and B, natural killer cells (NK cells) and endothelial cells [52]. CAFs, TAMs and MDSCs have a crucial role in cancer cell proliferation. Estrogen signaling is also known to play an important role in the regulation of the immunological response [6]. Their role is also visible in the TME. Both ERs and aromatase, which is a key enzyme in the production of estrogens, are expressed in cells that belong to the TME [54]. For example, expression of ERα and ERβ was observed in CAFs and TAMs in local TME of ovarian cancer [54]. The interaction of the cells in the organisms is mostly tissue-specific and depends on many factors. However, based on the literature survey, many of the effects stimulated by compartments of TME are mediated via PI3K/MAPK/Akt pathways, which are described as well-known ER-mediated pathways. Epithelial ovarian cancer (EOC) is in some kind unique among other solid tumors in the context of TME since tumor cells migrate from the primary tumor to create malignant ascites in the peritoneal cavity [55]. Malignant ascites also have TME and ascite-derived tumor cells occur as single floating cells or also as spheroids [55]. It is generally known that microRNAs (miRNA) are short, non-coding RNAs that regulate gene expression [56,57]. Their dysregulation has been observed in most types of cancer including ovarian cancer. Recently, it has been proposed that they also may have an influence on TME [56,57].

### 5.1. Cancer-Associated Fibroblasts (CAFs)

CAFs are the most predominant stromal cells that create TME. Zhang et al. observed that an increased amount of CAFs was in EOC than in benign tumors [52]. Moreover, CAFs isolated from EOC lesions were able to increase the invasion and migration of ovarian cancer cells [58,59]. Interestingly, Jin et al. showed that collapsin response mediator protein-2 (CRMP2) participates in these modulations through activation of the hypoxia-inducible factor (HIF)-1α–glycolysis signaling pathway [60]. CAFs relieve substances like chemokines and growth factors, which then stimulate pathways associated with tumor growth and progression [61]. Thongchot et al. showed that interleukin-8 (IL-8) released from CAFs increases the migration of ovarian cancer cells [62]. Similarly, fibroblast growth factor-1 (FGF-1) released from CAFs increased the growth of SKOV3 cells via the FGFR4/MAPK/ERK pathway [63]. In turn, Zhang et al. showed that CAFs induce EMT in OC cells via the Wnt/β-catenin pathway. Interestingly, it was also shown that progranulin (PGRN) stimulates the proliferation and invasion of ovarian cancer cells, indirectly via CAFs [64]. A similar observation has been made for TGF-β [65]. Wu et al. observed that collagen type XI alpha 1 (COL11A1) is upregulated in CAFs [66]. Moreover, they also noticed that its modulation may have an important role in the biology of OC, resulting in decreased invasiveness and tumor formation, giving hope that COL11A1 may have a key role in the future in the treatment of OC with elevated levels of TGF-β3 [66]. Yue et al. noticed that CAFs increase the metastasis character of ovarian cancer cells via the PI3K/Akt pathway [67]. Additionally, increased expression of E2—the responsive gene—was observed in CAFs compared to normal fibroblasts [68,69]. Kim et al. presented that expression of GLIS1 (Glis Family Zinc Finger 1) is increased both at gene and protein levels in CAFs. Moreover, the knockdown of GLIS1 decreased migration and invasion of ovarian cancer cells, suggesting that this factor may have an important role in the progression of OC [70]. It was observed that miRNAs in CAFs may affect their reprogramming. In the ovarian cancer microenvironment, and more specifically in the context of CAFs, miR-214 and miR-155 have been described at low and high expression, respectively [71]. It was further demonstrated that by disrupting their expression, it was possible to reduce the growth and metastasis of ovarian cancer through loss of the CAF-like phenotype, which seems to be very important in ovarian cancer treatment [71].

### 5.2. Tumor-Associated Macrophages (TAMs)

It is generally known that macrophages belong to phagocytic cells and that they regulate immunological response. Nevertheless, it has also been shown that they may regulate the invasion and metastasis of cancer cells, mainly because they can create an inflammatory environment, which may stimulate mutations, growth, proliferation, metastases and many others [72]. In primary OC as well as in ascites, the main population of immune cells consists of macrophages. They may originate from embryonic yolk sacs and bone-marrow-derived monocytes. When macrophages (M0) are recruited to TME, dependent on stimuli, they differentiate into the M1 or M2 subtype [73]. M1 macrophages inhibit the progression of cancer via the secretion of cytokines like IL-12, TNFα or IFNγ [73]. In turn, M2 macrophages stimulate the proliferation of cancer cells via the secretion of matrix metalloproteinases, IL-4, IL-5, IL-6 and other factors like VEGF [73]. In the OC microenvironment, TAMs mainly occur as the M2 phenotype, highly expressing scavenger receptor class B (CD163) and the mannose receptor (MR, CD204) [55]. Despite chemokines and cytokines that are well known to modulate M0 macrophage polarization, in recent years, increasing evidence indicates that microRNAs may also play an important role. Ying et al. showed that miR-222-3p secreted by OC cells stimulates the polarization of M0 cells into M2 via the SOCS3/STAT3 pathway [74]. MiRNA-940, miRNA-200b, miRNA-181c-5p and miRNA-141-3p have also been found to promote the M2-like phenotype [75,76,77,78]. In turn, Jiang et al. showed that miR-217 inhibits polarization into M2 cells by affecting IL-6 and the JAK3/STAT3 signaling pathway [79]. Both cancer cells and cells belonging to the TME secrete substances that cause their mutual interaction. Earlier, we indicated substances secreted by cancer cells that affect the polarization of macrophages. Nevertheless, polarized macrophages also secrete substances that can further affect cancer cells. It has been shown that M2 macrophages increase the proliferation and progression of OC [80]. Steitz et al. showed that TAMs derived from ascites promote invasion of HGSC via transforming growth factor beta-induced (TGFBI) protein and tenascin C (TNC) [80]. Zeng et al. presented that M2-like macrophages secrete epidermal growth factor (EGF) and thus affect EGFR-ERK signaling, leading to the progression of OC [81]. Ke et al. showed that TAMs increase the invasion of OC cells via the TLR signaling pathway and its downstream nuclear factor NFκB and microtubule-associated proteins’ (MAPs) kinases [82]. A co-culture of SKOV3 cells with macrophages resulted in increased migration and invasion [83,84]. It also increased the expression of NFκB, CXCL16 and CXCR6 and also affected the PI3K/Akt signaling pathway [83]. 

### 5.3. Myeloid-Derived Suppressor Cells (MDSCs)

**Figure 3:**
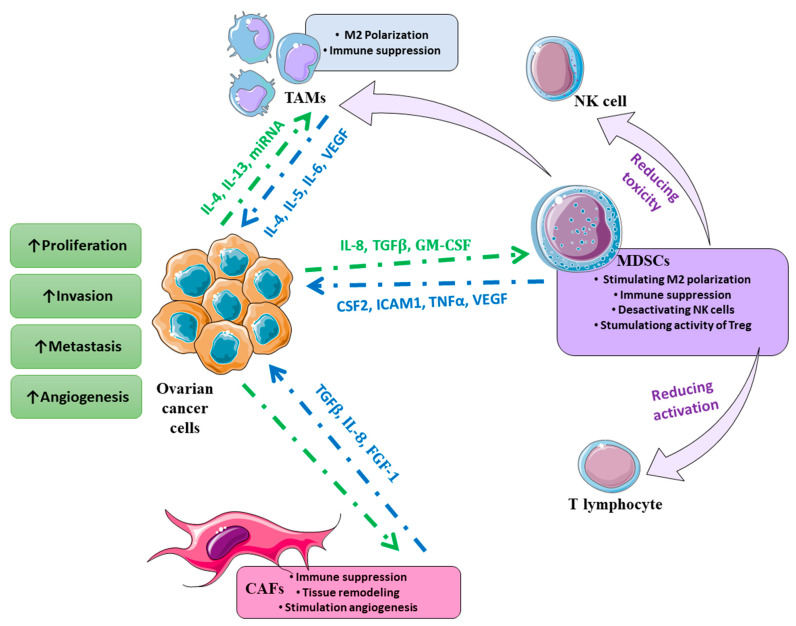
Schema presenting the interaction of the compartments of TME that stimulate ovarian cancer. TAMs—Tumor-Associated Macrophages, MDSCs—Myeloid-Derived Suppressor Cells, CAFs—Cancer-Associated Fibroblasts. The graphical illustration was prepared by using images from Servier Medical Art by Servier. Minor modifications were made (e.g., the color of the stock images) (https://smart.servier.com/smart_image/, accessed on 21 August 2023).

MDSCs are described as a heterogeneous population of cells of a myeloid origin in various stages of differentiation without mature myeloid markers [85]. It was observed that MDSCs are present in the peripheral blood derived from women with EOC [86]. In normal conditions, immature myeloid cells (IMCs) differentiate into mature forms like granulocytes, macrophages or dendritic cells [85]. Pathological conditions result in an expansion of IMCs due to the blocking of the differentiation and making them MDSCs. Interestingly, it was shown that obesity also has an impact on MDSCs and ovarian cancer [87]. Yang et al. showed that in obese mice, the proportion of MDSCs in peripheral blood was higher than in healthy mice [87]. MDSCs present higher expression of immune suppressive factors like *arginase 1* (*ARG1*), and inducible nitric oxide synthase (iNOS). A characteristic is also increased expression of CD33+ on their surface and increased production of nitric oxide (NO) and reactive oxygen species (ROS) [85,88]. MDSCs have immune suppressive functions by directly affecting T and NK cells and also stimulating angiogenesis, proliferation, invasion and metastases of the tumor [89,90,91]. Cui et al. showed that MDSCs increased cancer stemness via inhibition of T cell activation and affection of the expression of miRNA101 and the corepressor gene C-terminal binding protein-2 (CtBP2) [91]. Li et al. also observed that MDSCs enhance the stemness of EOC cells [86]. The authors also verified genes that were modulated during a co-culture of MDSCs and SKOV3 cells and they observed that colony-stimulating factor 2 (CSF2), intercellular adhesion molecule 1 (ICAM1), baculoviral IAP repeat-containing 3 (BIRC3), TNFα-induced protein 3 (TNFAIP3) and interleukin-32 (IL-32) increased significantly [86]. Zheng et al. showed that upregulation of miRNA-211 decreased MDSC proliferation and affected pathways like NF-κB, PI3K/Akt and STAT3 [92]. Taki et al. presented that knockdown of Snail in mouse OC cells resulted in an inhibited growth and decreased amount of MDSC cells. Snail was also described as a factor that recruits MDSC in the OC environment via regulation of the expression of the interleukin-8 receptor, beta (CXCR2) ligands [93]. Vascular endothelial growth factor (VEGF) has also been proposed as a factor that recruits MDSCs [94]. Nevertheless, usage of anti-VEGF therapy increases granulocyte–monocyte colony-stimulating factor (GM-CSF) expression and thus recruits MDSCs; therefore, Horikawa et al. suggested that targeting GM-CSF may help overcome resistance to anti-VEGF therapy [95]. Summarized effects of TAMs, CAFs and MDSCs are presented in Figure 3. 

## 6. Conclusions

In recent years, there is more and more evidence that estrogens play a key role in the formation of all kinds of hormone-dependent cancers, such as breast, prostate or ovarian cancer. From this review of the literature, it is clear that estrogens or estrogen-like compounds, acting through the ERs, regulate various cellular processes such as proliferation, EMT, invasiveness, differentiation and inflammation in ovarian cancer cells. It seems that all receptors play a very important role in the process of ovarian carcinogenesis, and keeping their proportions in a physiological state is necessary to stay healthy. Moreover, it seems that estrogens are also necessary for the regulation of the TME because most of the estrogen-related pathways are disrupted and thus contribute to the pro-tumor function of the TME. In conclusion, estrogens and estrogen-like compounds play an important role in ovarian carcinogenesis through direct or indirect involvement in molecular mechanisms stimulating the growth and proliferation of cancer cells. Further studies are needed to elucidate all molecular mechanisms of estrogen signaling in ovarian cancer cells and the components of TME, due to their crucial role in cancer progression and therapy. Enriched knowledge about estrogens, estrogen receptors and the tumor microenvironment may encourage us to look for new therapeutic options for patients.

## 7. Limitations of the Review

During the review associated with tumor microenvironment (TME), we focused on cells that are associated with ovarian cancer proliferation; nevertheless, it should be emphasized that more types of cells are included in TME and they may also be worth reviewing.

## Figures and Tables

**Table 1:** Summarized information about compounds and their influence on ER expression in ovarian cancer cells. ERs—estrogen receptors, ERα—Estrogen receptor α, ERβ—Estrogen receptor β, ↑—upregulation, ↓—downregulation.

Substance	Concentration	Time of Exposition (h)	Cell Line	ERs	Literature
Histamine	50 ng/mL	48	OVCAR3	↑ERα ↓ERβ	[44]
Aconitine	100, 200 and 400 µg/mL	24	A2780	↑ERβ	[49]
Genistein	10 and 50 µM	24	SKOV3, OVCAR3, A2780CP	↑ERβ	[40]
Daidzein	10 and 50 µM	24	SKOV3, OVCAR3, A2780CP	↑ERβ	[40]
Bisphenol A	10 µM	6 and 24	BG-1	↑ERα	[41,43]
Cadmium Chloride	0.01 µM	24	OVCAR3	↑ERα ↑ERβ	[51]

## Data Availability

Not applicable.
